# Serum antibodies against mimotopes of Merkel cell polyomavirus oncoproteins detected by a novel immunoassay in healthy individuals and Merkel cell carcinoma patients

**DOI:** 10.1111/1751-7915.14536

**Published:** 2024-10-25

**Authors:** Chiara Mazziotta, Giada Badiale, Christian Felice Cervellera, Giulia Tonnini, Milena Oimo, Antoine Touzé, Françoise Arnold, Stefania Zanussi, Ornella Schioppa, Giuseppe Fanetti, Mauro Tognon, Fernanda Martini, John Charles Rotondo

**Affiliations:** ^1^ Department of Medical Sciences University of Ferrara Ferrara Italy; ^2^ Center for Studies on Gender Medicine, Department of Medical Sciences University of Ferrara Ferrara Italy; ^3^ “Biologie Des Infections à Polyomavirus” Team UMR INRAE 1282 ISP, University of Tours Tours France; ^4^ Immunopathology and Cancer Biomarkers Unit, Centro di Riferimento Oncologico (CRO) Aviano IRCCS, National Cancer Institute Aviano Italy; ^5^ Infectious Diseases and Tumors Unit, Department of Medical Oncology, Centro di Riferimento Oncologico (CRO) IRCCS, National Cancer Institute Aviano Italy; ^6^ Division of Radiotherapy Centro di Riferimento Oncologico (CRO), IRCCS Aviano Italy; ^7^ Laboratory for Technologies of Advanced Therapies (LTTA) University of Ferrara Ferrara Italy

## Abstract

Merkel cell polyomavirus (MCPyV) is the foremost causative factor of Merkel cell carcinoma (MCC), a rare yet highly aggressive skin cancer. Although the evaluation of circulating IgG antibodies against Merkel cell polyomavirus (MCPyV) LT/sT oncoproteins is clinically useful for MCC diagnosis/prognosis, a limited number of assays for identifying such antibodies have been developed. Herein, a novel indirect immunoassay with synthetic epitopes/mimotopes of MCPyV oncoproteins was computationally designed and experimentally validated on control sera and sera from healthy individuals and MCC patients. Upon computational design of five synthetic peptides, the performance of the immunoassay in detecting anti‐oncoprotein IgGs in MCPyV‐positive and ‐negative control sera was evaluated. The immunoassay was afterwards extended on sera from healthy individuals, and, for longitudinal analysis, MCC patients. Performance properties such as sensitivity and specificity and positive/negative predictive values were adequate. Receiver‐operating characteristic (ROC) curves indicated that the areas under the curves (AUCs) were within the low/moderately accurate ranges. Immunoassay was repeatable, reproducible and accurate. As expected, the serum anti‐oncoprotein IgG prevalence in healthy individuals was low (2%–5%). Anti‐oncoprotein IgGs slightly increased when MCC patients experienced partial tumour remission and/or stable disease, compared to baseline. Our data indicate that the newly developed immunoassay is reliable for detecting circulating anti‐oncoprotein IgGs both in healthy individuals and MCC patients.

## Graphical abstract

In this study, a novel immunoassay for detecting human antibodies against Merkel cell polyomavirus LT and sT oncoproteins was developed. Upon computational design of five synthetic peptides, the immunoassay performance was evaluated in (i) control human sera and (ii) serum samples from healthy individuals and Merkel cell carcinoma patients.

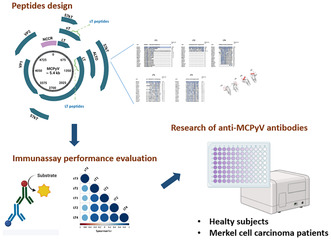

## INTRODUCTION

Merkel cell polyomavirus (MCPyV) is a small DNA virus, that drives the development of the rare but highly aggressive skin cancer Merkel Cell Carcinoma (MCC) (Mazziotta, Cervellera, Badiale, et al., 2023; Mazziotta, Cervellera, Lanzillotti, et al., 2023; Mazziotta et al., 2024; Samimi et al., 2020). MCPyV infection is near‐ubiquitous and asymptomatic in humans. Varying rates of MCPyV serology have been reported across different healthy populations (Silling et al., 2022), according to the immunological study considered (Carter et al., 2009; Nicol et al., 2013; Van Der Meijden et al., 2013; Xue & Thakuria, 2019). However, recent data concordantly indicate that the MCPyV infection occurs in early childhood (Mazziotta, Lanzillotti, Govoni, et al., 2023).

MCPyV genome is a circular and double‐strand DNA containing the regulatory non‐coding control region (NCCR), the replication origin and two bidirectional promoters controlling the expression of early and late genes (Decaprio, 2021; Wendzicki et al., 2015). During the life cycle, DNA enters the nucleus and expresses the early genes large tumour (LT) antigen and small tumour antigen (sT), which in turn stimulate the viral DNA replication. Transcriptional activation of the late genes viral protein 1 (VP1) and viral protein 2 (VP2) follows viral DNA replication initiation and leads to the production of the viral capsid.

Upon primary infection, MCPyV triggers a natural immune response that persists lifelong in immunocompetent hosts, effectively limiting viral replication (Liu et al., 2016). Conditions of early host immune response impairment promotes the MCPyV replication activity, leading to the viral DNA integration into the host genome, which is paralleled with the clonal expression of truncated LT/sT oncoproteins (Tognon et al., 2024). These processes trigger MCC carcinogenesis and drive disease progression (Liu et al., 2016). As a result, MCPyV‐positive MCCs persistently express LT and sT oncoproteins while capsid proteins are rarely synthesized. Persistent LT/sT expression induces immune system stimulation, increasing circulating IgGs to viral oncoproteins. In this context, assessing the serological response against MCPyV oncoproteins rather than viral capsid proteins in patients during therapy or individuals at risk of developing MCPyV‐driven MCC could hold clinical significance (Samimi et al., 2016).

The identification of robust biomarkers for MCC diagnosis and prognosis is an unsolved clinical issue (Paulson et al., 2010, 2017; Samimi et al., 2016). Moreover, the lack of clinically useful prognostic markers limits the development of effective models for MCC prognostication (Mazziotta, Cervellera, Lanzillotti, et al., 2023). Evaluating circulating antibodies against MCPyV antigens might present clinical utility (Paulson et al., 2010, 2017; Samimi et al., 2016). Despite MCPyV oncogenicity is primarily mediated by LT/sT (Prado et al., 2018), the majority of current immunological methods have been designed for the detection of antibodies recognizing MCPyV VP1/2. However, there is a limited clinical applicability for exploiting anti‐capsid antibodies as biomarkers, especially for early tumour detection, as capsid proteins are highly visible to the humoral immune system of immunocompetent individuals (Carter et al., 2009; Van Der Meijden et al., 2013; Xue & Thakuria, 2019), while virus‐positive MCCs do not express VPs (Touzé et al., 2011). The investigation of anti‐oncoprotein antibodies might circumvent this drawback. However, a restricted number studies have been conducted for this purpose, although with promising results (Paulson et al., 2010, 2017; Samimi et al., 2016). Methods developed for detecting anti‐MCPyV antibodies cumulatively rely on the use of either recombinant VP or LT proteins, as antigens (Li et al., 2015). These methods are time consuming as the production of viral recombinant proteins encompasses multiple difficult steps, including the gene coding sequence cloning and protein synthesis with bacteria systems in vitro. Then, recombinant proteins should be isolated, quantified and assayed (Nicol et al., 2013; Samimi et al., 2016; Touzé et al., 2010). An additional limitation of recombinant proteins is the potential generation of cross‐reactions among viruses from the same family with a certain degree of homology (Samimi et al., 2016; Touzé et al., 2010). Affordable, rapid and specific immunoassays for the identification of anti‐oncoprotein antibodies are therefore yet lacking.

We have previously designed a reliable method with synthetic peptides mimicking MCPyV capsid proteins for the detection of anti‐capsid antibodies, without using viral recombinant proteins (Mazziotta, Lanzillotti, Govoni, et al., 2021, 2023; Mazziotta, Lanzillotti, Torreggiani, et al., 2021; Mazziotta, Pellielo, Tognon, et al., 2021). To address need of specific methods for detecting antibodies against MCPyV oncoproteins, we have computationally designed and experimentally developed and validated in clinical samples such as sera from healthy individuals with unknown MCPyV serology and MCC patients, a novel immunoassay with synthetic peptides mimicking MCPyV LT and sT antigens.

## RESULTS

### Sequence analyses

**FIGURE 1:**
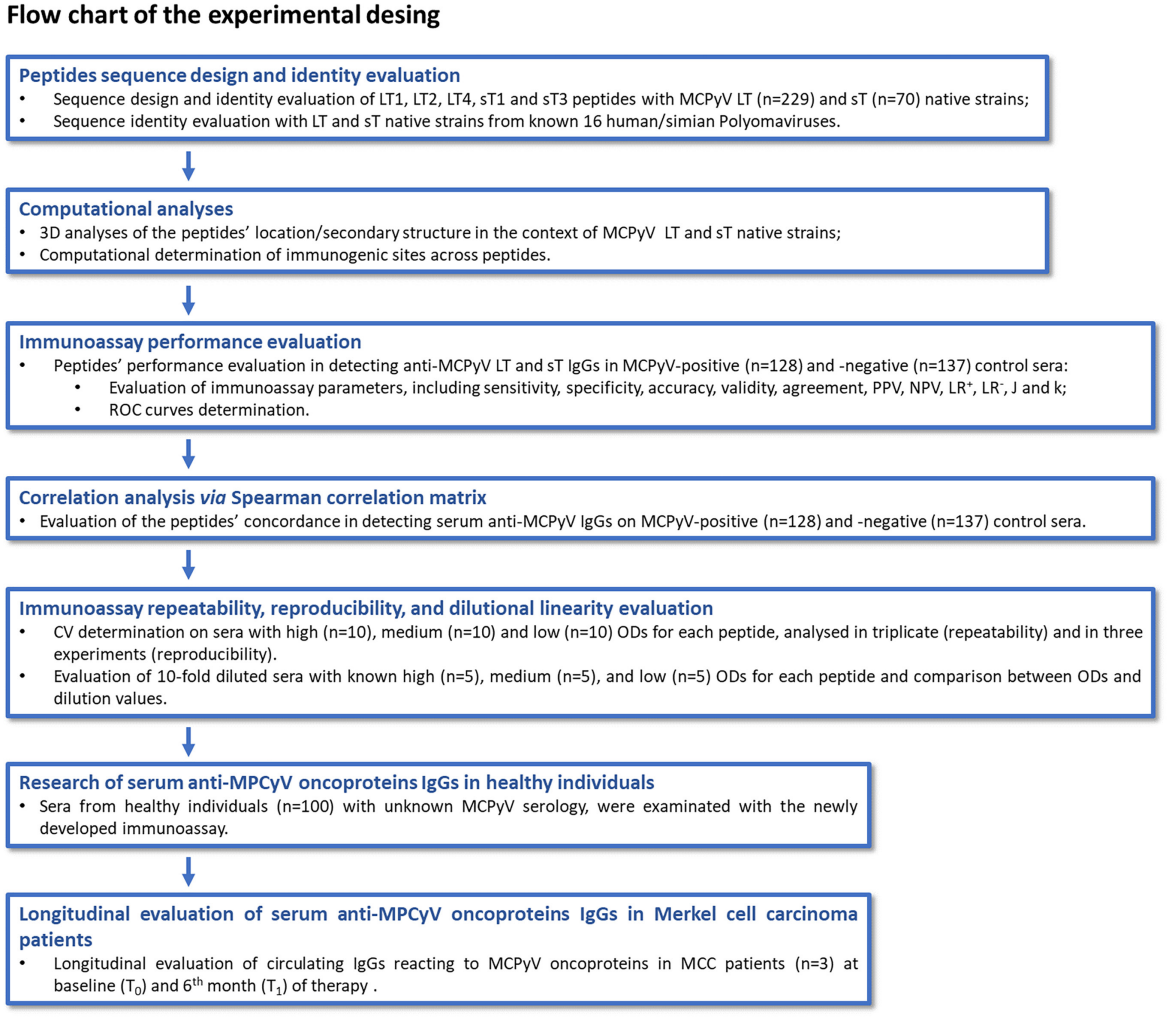
Flow chart of the immunoassay developed and validation. The immunoassay was development and validated for the detection of circulating anti‐Merkel cell polyomavirus (MCPyV) Large T (LT) and small T (sT) oncoproteins IgG antibodies. 3D, Three‐dimensional; CV, coefficient of variation; J, Youden's Index;k, Cohen's Kappa value; LR^+^ and LR^−^, positive and negative likelihood ratios; MCC, Merkel cell carcinoma.; NPV and PPV, negative and positive predictive values; OD, optical density; ROC, receiver operating characteristic.

**FIGURE 2:**
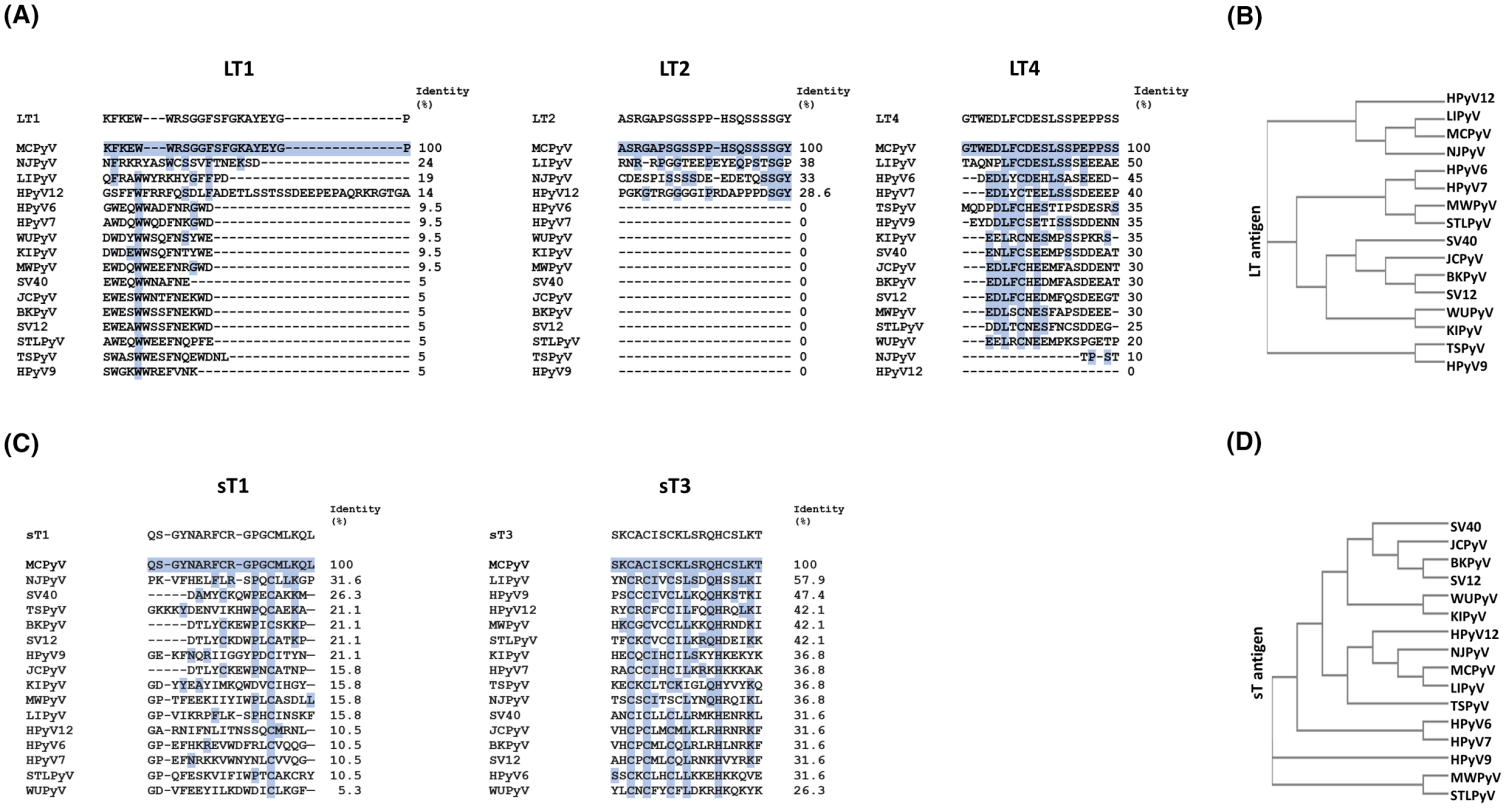
Amino acid sequences of LT1/2/4, sT1/3 peptides, and polyomaviruses phylogenetic trees. (A, C) Identity between Merkel cell polyomavirus (MCPyV)‐specific LT1/2/4 and sT1/3 peptides and LT and sT native strains from human/simian polyomaviruses (PyVs). All LT and sT peptides were selected based on their low homology with corresponding a.a. sequences from LT and sT from known human/simian PyVs. LT and sT a.a. sequences/IDs were obtained from (https://www.ncbi.nlm.nih.gov/protein/) and aligned with Clustal Omega software. (B, D) PyVs phylogenetic trees according to LT and sT from human/simian PyVs comparisons.

The experimental design is depicted in Figure 1. Upon computational design, it was assessed the identity between LT1/2/3 and sT1/3 a.a. sequences and corresponding LT/sT native strains of 16 PyVs, including MCPyV (Figure 2A,B). The LT1 a.a. sequence was 100% identical to the corresponding LT from 229 MCPyV isolated strains. The LT2 a.a. chain was 100% identical to that of the corresponding LT from 226 MCPyV isolated strains while being different for 1 a.a. from A0A0D3LRX9, X5FLV8, and E2GHQ3 strains. The LT4 a.a. sequence was 100% concordant to that of the corresponding LT from 227 MCPyV isolated strains, and 95% (19/20 a.a.) concordant with that of A0A0D3LRZ4 and G8FSZ3 strains. The sT1 and sT3 a.a. chains were 100% identical to that of the sT from all 70 isolated MCPyV strains. In a few cases, the identity of all peptides with LT/sT native strains from other 15 PyVs was between 55 and 32%, and below 32% in the majority of cases (Figure 2C,D). Peptides also differ from LT/sT native strains from 15 PyVs, due to the presence (or absence) of additional a.a. residues within their sequence.

### Three‐dimensional analyses

**FIGURE 3:**
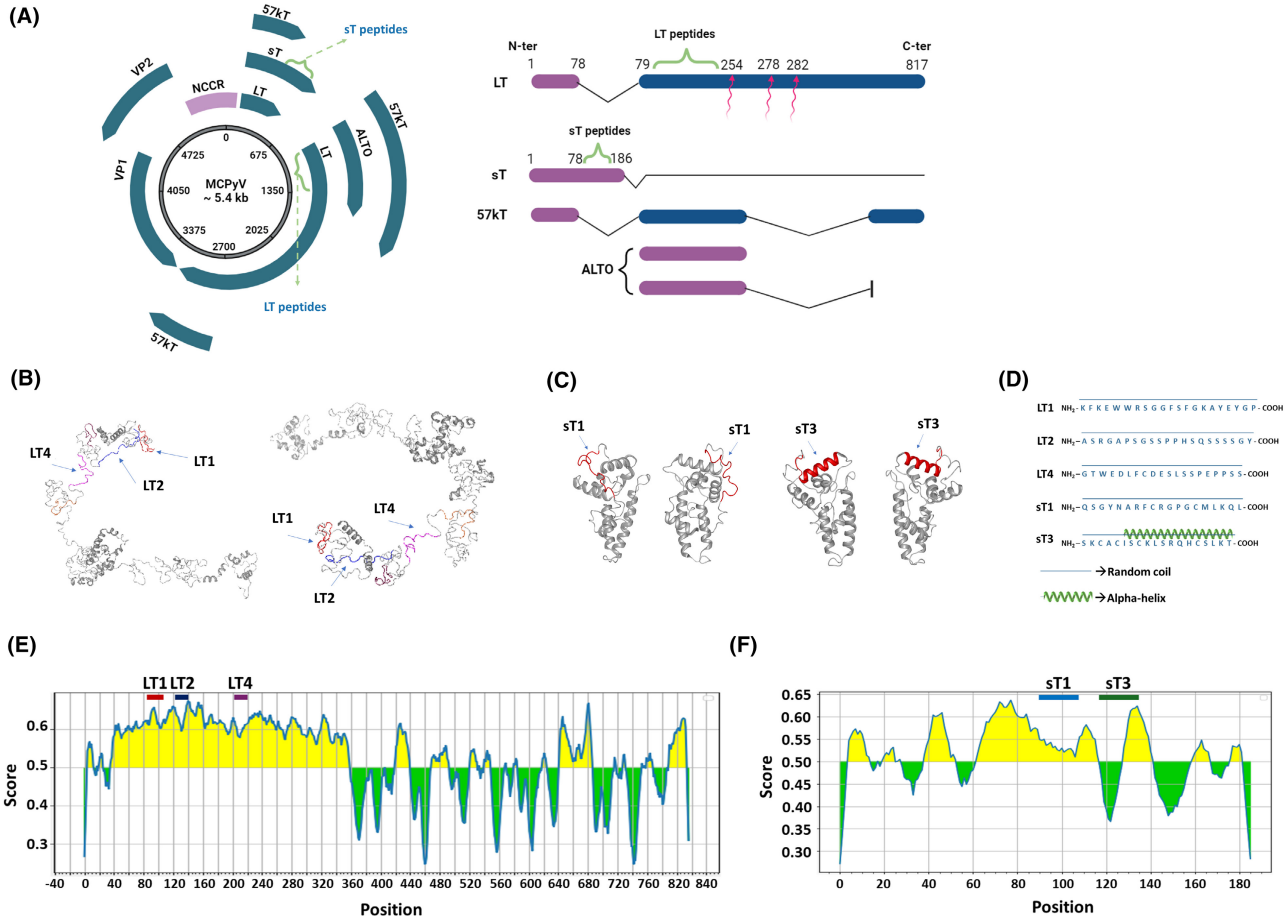
Circular structure of Merkel cell polyomavirus (MCPyV) genome, linear maps of early genes and MCPyV Large T (LT) and small T (sT) peptides localization on the genome and MCPyV proteins partial structures visualizations and B‐cell linear epitopes prediction of MCPyV LT and sT amino acid sequences. (A) MCPyV is a small double‐stranded DNA tumour virus with a circular genome of 5387 bp (right panel). The MCPyV genome is divided in early and late coding regions, that represent the two transcriptional units, and by the non‐coding control region (NCCR) which contains a bidirectional promoter and the viral origin of replication (right panel). The late region codifies for two viral capsid proteins named VP1 and 2. LT1/2/4 and sT1/3 peptides are specifically localized across LT and sT coding sequences, respectively (right panel). Several transcripts are encoded from the early region by alternative splicing comprising LT and sT, 57 kT antigen (57 kT), small T antigen (sT), and the alternative frame of the large T open reading frame (ALTO) (left panel). Red arrows represent truncating sites within the LT amino acid sequence (left panel). LT and sT peptides localization (green) within LT and sT amino acid sequences (right panel). (B, C) Partial view of MCPyV LT1/2/4, in red, blue, and pink, respectively, and sT1/3 peptides, in red, within the three‐dimensional (3D) renderings of LT and sT proteins. LT and sT representations are based on a structural prediction obtained using DNASTAR software. (D) Computational analyses indicate that all the peptides show several random coiled domains. The peptide sT3 also shows an alpha helix domain (in green). (E, F) Graphical representations of predicted B‐cell epitopes (in yellow) and non‐epitopes (green) of LT (panel E) and sT (panel F) a.a. sequences. LT1/2/4 and sT1 peptides were located within predicted B‐cell epitopes. sT3 peptide was partially located within predicted epitopes. Prediction with default values was performed in BepiPred Linear Epitope Predictor 2.0.

Peptides' location/secondary structure in the context of MCPyV LT/sT were determined computationally (Figure 3A,D). LT1, LT2, and LT4 correspond to a.a. 89–109, 125–145 and 207–226, respectively, of the LT reference strain B6DVW7_9POLY LT, while sT1 and sT3 lie within a.a. 96–114 and 117–135 of the sT reference strain B0G0V7_9POLY. The three‐dimensional (3D) rendering of LT1/2/3 and sT1/3 mapped onto the inferred MCPyV LT and sT native structures, respectively, indicated that these regions are located on protein surfaces (Figure 3B,C). LT1/2/3 and sT1/3 a.a. sequences were characterized toward a stable secondary structure formation, and by the presence of several random coiled domains. Moreover, sT3 forms a stable secondary structure from a.a. 122 to 135, i.e., _122_ISCKLSRQHCSLKT_135_, where an alpha‐helix domain is found, surrounded by two random coil structures (Figure 3D).

### Computational determination of immunogenic sites

BepiPred 2.0 identified a total of 21 and 9 B‐cell linear epitopes within MCPyV LT and sT native strains, respectively. Notably, LT1/2/4 and sT1 peptides resulted in being located within predicted B‐cell epitopes with mean scores of 0.628, 0.637, 0.605, and 0.54, respectively (Figure 3E,F). sT3 peptide was partially located within predicted epitopes, with 7/19 a.a. identified as epitopes with an overall mean score of 0.5.

### Immunoassay performance parameters evaluation

The immunoassay performance in detecting serum anti‐MCPyV oncoproteins IgGs in MCPyV‐positive/−negative control sera was evaluated (Table 1). Peptides showed sensitivities and specificities ranging from 72% to 82.81% and 60.5% to 74.8%, respectively, while PPVs and NPVs ranged from 65.7% to 76.3% and 67.3% to 80.8%. Immunoassay validity and accuracy ranged from 73.22% to 77.5% and 73.21% to 77.35%, respectively, for LT1/2/4, and 66.25% to 77.64% and 66.40% to 77.65%, for sT1/3. Moreover, J, LR^+^ and LR^−^ ranged from 0.46 to 0.55, 2.52 to 3.09 and 0.25 to 0.36, respectively, for LT1/2/4, and 0.33 to 0.55, 1.82 to 3.19 and 0.26 to 0.46, for sT1/3. Ef was 77.36% with *κ* = 0.548 for LT1, 73.2% with *κ* = 0.463 for LT2, and 74.71% with a *κ* = 0.496, for LT4. Similarly, the Efs of sT1 and sT3 were 77.7% and 66.39%, with *κ* = 0.552 and *k* = 0.325 for sT1 and sT3, respectively.

**TABLE 1:** Merkel cell polyomavirus‐based immune assay performance parameters.

	LT1	LT2	LT4	sT1	sT3
Sensitivity (Se)	81.30% (73.4–87.6)	73.44% (64.9–80.9)	82.81% (75.1–88.9)	80.50% (72.5–86.9)	72.00% (63.3–79.7)
Specificity (Sp)	73.72% (65.5–80.8)	73.00% (75.0–80.2)	67.15% (62.0–74.9)	74.80% (66.3–82.0)	60.50% (51.1–69.3)
Accuracy	77.35% (71.8–82.3)	73.21% (67.5–78.4)	74.72% (69.0–79.8)	77.65% (72.0–82.6)	66.40% (60.1–72.3)
Likelihood ratio (LR^+^)	3.09 (2.31–4.14)	2.72 (2.03–3.65)	2.52 (1.96–3.24)	3.19 (2.34–4.36)	1.82 (1.42–2.34)
Likelihood ratio (LR^−^)	0.25 (0.18–0.37)	0.36 (0.27–0.49)	0.26 (0.17–0.38)	0.26 (0.18–0.38)	0.46 (0.34–0.64)
Positive predictive value (PPV)	74.30% (68.3–79.5)	71.80% (65.4–77.3)	70.20% (64.7–75.2)	76.30% (70.2–81.5)	65.70% (59.9–71.0)
Negative predictive value (NPV)	80.80% (74.3–86.0)	74.63% (68.4–80.0)	80.70% (73.8–86.2)	79.17% (72.5–84.6)	67.30% (60.0–73.8)
Validity	77.50%	73.22%	75.00%	77.64%	66.25%
Youden's Index (*J*)	0.55	0.46	0.50	0.55	0.33
Overall efficiency (Ef)	77.36%	73.20%	74.71%	77.70%	66.39%
Cohen's Kappa value (*κ*)	0.548	0.463	0.496	0.552	0.325

*Note*: Immunoassay diagnostic performance parameters obtained by testing MCPyV‐positive (*n* = 128) and ‐negative (*n* = 137) control sera. Numbers surrounded by parentheses for sensitivity, specificity, accuracy, LR^+^, LR^−^, PPV, and NPV parameters represent the 95% Confidence Interval (CI). The following conditions were considered: true positive (TP), false positive (FP), true negative (TN) and false negative (FN). The assay performance was assessed computing numerous set‐up parameters, as follows: Sensitivity, specificity, positive predictive value (PPV), negative predictive value (NPV), validity, accuracy, overall efficiency (Ef), Youden's Index, positive and negative likelihood ratios (LR^+^, as Se/[1‐Sp] and LR^−^, as [1‐Se]/Sp, respectively) and Cohen's Kappa value (*k*).

### Immunoassay receiver operating characteristic (ROC) curves and correlation analysis

**FIGURE 4:**
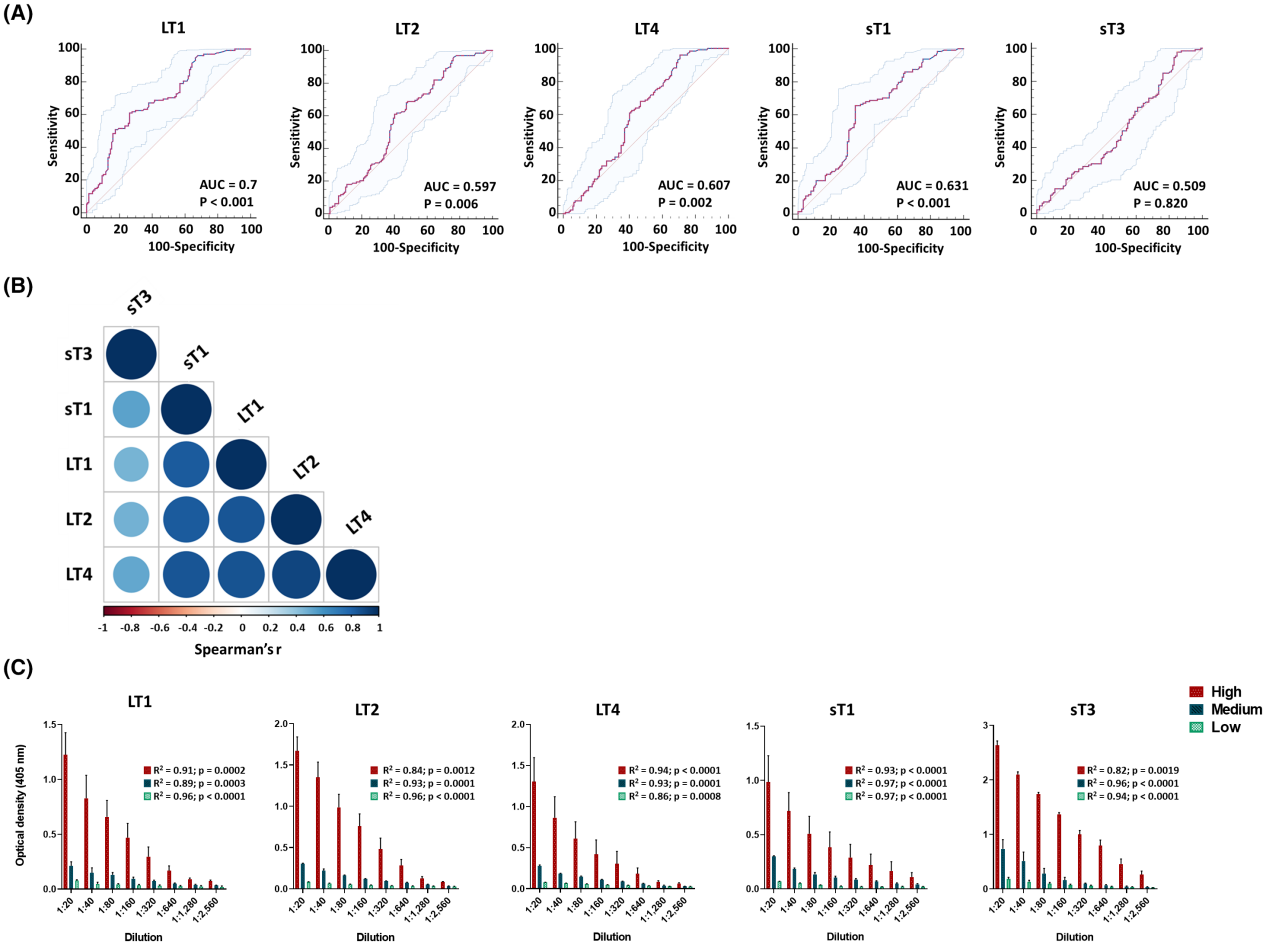
Receiver‐operator characteristic (ROC) curves, correlation, and dilutional linearity of optical density (OD) values obtained using Merkel cell polyomavirus (MCPyV) LT1/2/4 and sT1/3 peptides. (A) ROC curves were built based OD obtained on MCPyV‐positive (*n* = 128) and ‐negative (*n* = 137) control sera, for both MCPyV LT1/2/4 and sT1/3 peptides. AUC values range between 0.5 and 1, interpreted as follows: Non‐informative/worthless test, AUC = 0.5; low accurate, 0.5 < AUC ≤0.7; moderately accurate, 0.7 < AUC ≤ 0.9; highly accurate, 0.9 < AUC < 1; perfect, AUC = 1 60. The values for the area under the ROC curve (AUC) were 0.7 (95% CI: 0.641 to 0.754), 0.597 (95% CI: 0.535 to 0.656), 0.607 (95% CI: 0.545 to 0.666) for LT1, LT2 and LT4, respectively. The values for the AUC were 0.631 (95% CI: 0.569 to 690) and 0.509 (95% CI: 0.444 to 0.573) for sT1 and sT3 peptides, respectively. The diagonal line shows an AUC value of 0.5 which is representative of a worthless test. The difference between AUCs for the peptides LT1/2/4 and sT1 peptides resulted statistically significantly different from that of a worthless test (AUC = 0.5, *p* < 0.01). (B) The concordance in ODs among the LT1/2/4, and sT1/3 peptides was evaluated on the entire set of MCPyV‐positive (*n* = 128) and ‐negative (*n* = 137) control sera using Spearman correlation matrix analysis. A strong concordance between LT1 and LT2 peptides was determined, as indicated by an r‐value of 0.83 and a *p* < 0.0001. A robust correlation was also noted between LT1 and LT4, with a Spearman r value of 0.86 and a *p* < 0.0001. The OD concordance between LT2 and LT4 peptides demonstrated a high level of correlation, as shown by an r of 0.9 and a *p* < 0.0001. Furthermore, the concordance between sT1 and sT3 peptides was moderate, with an r of 0.55 and a *p* < 0.0001. (C) OD response to serial dilutions (1:20, 1:80, 1:160, 1:320, 1:640, 1:1280 and 1:2560) of MCPyV‐positive sera presenting known high (*n* = 5), medium (*n* = 5), and low (*n* = 5) ODs. Each dilution was assayed in triplicate for each MCPyV LT1/2/4 and sT1/3 peptide, and ODs and sera dilutions were compared by linear regression analysis. High correlation between ODs and dilutions was found for all the LT and sT peptides with *R* ^2^ ranging from 0.8211 to 0.9791.

ROCs were computed based on ODs obtained on MCPyV‐positive/−negative control sera (Figure 4A). AUCs of LT1, LT2, LT4, sT1 and sT3 were 0.7, 0.597, 0.607, 0.631 and 0.509, respectively. AUCs of LT1, LT2 and LT4 and sT1 were significantly higher than that of a worthless test (AUC = 0.5, *p* < 0.01) and within the adequate reference range of 0.6–0.7 (Figure 4A). The OD concordance among peptides was evaluated on the entire set of control sera via Spearman correlation matrix analysis (Figure 4A–C). Concordances between LT1 and LT2 as well as LT1 and LT4 ODs were good, with r values of 0.83 and 0.86, respectively (*p* < 0.0001). Moreover, a good correlation was found between LT2 and LT4 ODs with an r of 0.9 (*p* < 0.0001). Concordance between sT1 and sT3 ODs were moderate with an r of 0.55 (*p* < 0.0001).

### Immunoassay precision and dilutional linearity evaluation

Repeatability and reproducibility data are depicted in Table 2. Repeatability CVs for the low, medium, and high ODs groups, for all peptides, ranged from 1.88% to 3.41%, 2.32% to 6.32% and 5.25% to 7.15%, respectively. Reproducibility CVs for the low, medium, and high ODs groups, for all peptides, ranged from 3.33% to 5.87%, 3.99% to 7.61% and 5.86% to 8.02%, respectively. Details on the dilutional linearity are reported in Figure 4C. A high correlation between ODs and dilutions for LT1, LT2 and LT4 was determined, with *R* ^2^s ranging from 0.89 to 0.91, 0.84 to 0.96 and 0.86 to 0.94, respectively (*p* < 0.01–< 0.001). A good correlation between ODs and dilutions for sT1 and sT3 was noted, with *R* ^2^s ranging from 0.93 to 0.97 and 0.82 to 0.96, respectively (*p* < 0.01–*p* < 0.0001).

**TABLE 2:** Intra‐assay and inter‐assay coefficients of variation of serum samples with high, medium and low optical density level.

	Optical density		
	Low	Medium	High
	(*n* = 10)	(*n* = 10)	(*n* = 10)
LT1			
Intra‐assay (*n* = 3)			
Mean	0.075	0.210	1.225
SD	0.012	0.039	0.077
CV (%)	2.17	3.28	5.45
Inter‐assay (*n* = 3)			
Mean	0.071	0.223	1.116
SD	0.205	0.068	0.13
CV (%)	3.33	5.19	5.86
LT2			
Intra‐assay (*n* = 3)			
Mean	0.080	0.306	1.670
SD	0.006	0.008	0.378
CV (%)	1.88	2.32	5.25
Inter‐assay (*n* = 3)			
Mean	0.097	0.315	1.615
SD	0.122	0.012	0.501
CV (%)	3.65	4.09	6.88
LT4			
Intra‐assay (*n* = 3)			
Mean	0.076	0.288	1.302
SD	0.120	0.025	0.125
CV (%)	2.05	4.02	5.88
Inter‐assay (*n* = 3)			
Mean	0.067	0.277	1.326
SD	0.032	0.079	0.311
CV (%)	3.76	3.99	5.77
sT1			
Intra‐assay (*n* = 3)			
Mean	0.069	0.294	1.222
SD	0.001	0.016	0.232
CV (%)	1.51	3.16	6.17
Inter‐assay (*n* = 3)			
Mean	0.073	0.327	1.151
SD	0.012	0.263	0.512
CV (%)	4.51	6.07	7.03
sT3			
Intra‐assay (*n* = 3)			
Mean	0.184	0.831	2.632
SD	0.062	0.362	0.166
CV (%)	3.41	6.32	7.15
Inter‐assay (*n* = 3)			
Mean	0.155	0.866	2.223
SD	0.102	0.201	0.387
CV (%)	5.87	7.61	8.02

*Note*: A total of 10 sera of known high ODs, i.e, OD > 0.55, >1.0, >0.65, >1.0 and >2.0 for LT1, LT2, LT4, sT‐1, and sT3 peptides, respectively, (ii) 10 sera of known medium ODs, i.e, 0.15 < OD < 0.55, 0.25 < OD < 1.0, 0.25 < OD < 0.65, 0.25 < OD < 1.0, 0.5 < OD < 2.0, for LT1, LT2, LT4, sT1, and sT3 peptides, respectively; (iii) 10 sera of known low ODs, i.e, OD < 0.15, <0.25, <0.25, <0.25 and <0.5 were selected for LT1, LT2, LT4, sT1, and sT3 peptides, respectively.

Abbreviations: CV, coefficient of variation; SD, standard deviation.

### Research of serum anti‐MCPyV oncoproteins IgGs in healthy individuals

The immunoassay was extended on sera from healthy individuals (*n* = 100). Sera reacting to LT1/2/4 reached an overall similar prevalence ranging from 2% to 3% (*p* > 0.05). All sera reacting to LT1/2/4 peptides, with exception of one, were from males. Median IgG ODs for LT1, LT2 and LT4 across positive sera was 0.5, 1.6 and 0.7, respectively. Sera reacting to sT1/3 had an overall similar prevalence of 5%–7% (*p* > 0.05). Among sera reacting to sT1/3, two were from females and six from males. Median IgG ODs for sT1 and sT3 across positive sera were 0.6 and 2.1, respectively. Although with (few) exceptions, the majority of sera concordantly reacted to each specific peptide and vice versa.

### Longitudinal evaluation of anti‐MPCyV oncoproteins IgGs in sera from Merkel cell carcinoma patients

**FIGURE 5:**
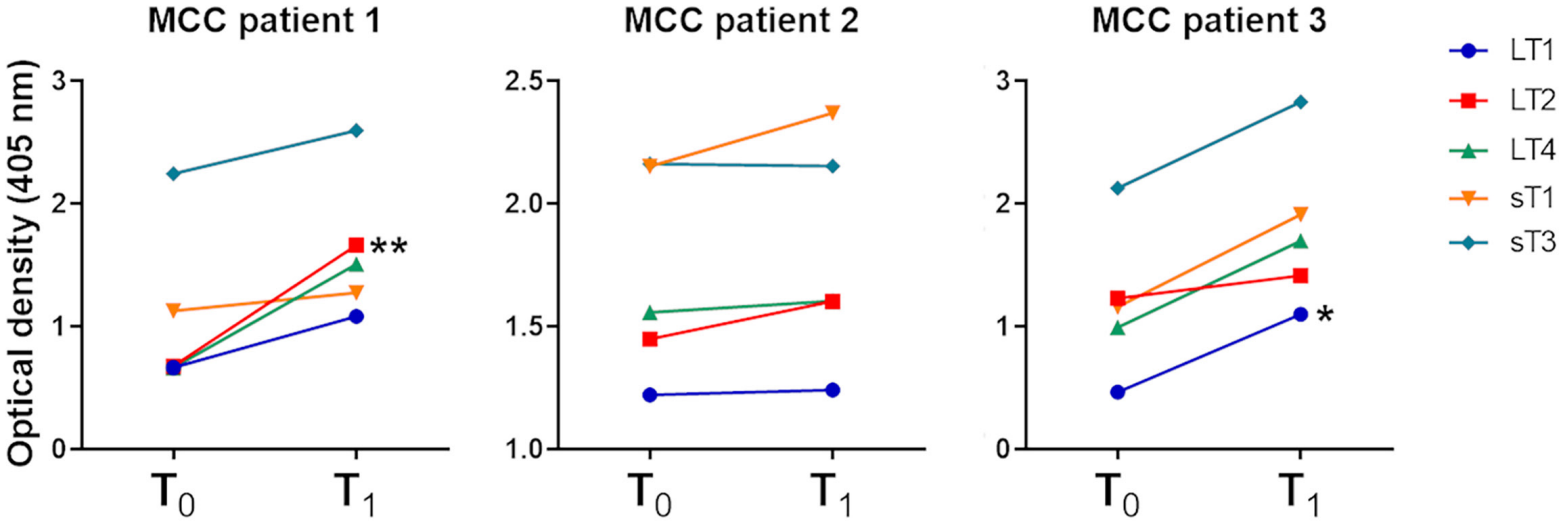
Longitudinal evaluation of serum anti‐MPCyV oncoproteins IgGs in Merkel cell carcinoma (MCC) patients. The anti‐MPCyV oncoproteins IgGs levels were evaluated in MCC patients (*n* = 3) at baseline (T_0_) and 6th month (T_1_) of therapy. All patients resulted positive with all tested LT1/2/4 and sT1/3 peptides. A significant increase in serum anti‐MCPyV IgG ODs from T_0_ (0.7) to T_1_ (1.7) was detected in patient 1 with LT2 peptide. Patient 3 showed a statistically significant increase in serum anti‐MCPyV IgG ODs from T_0_ (0.5) to T_1_ (1.1) with LT1. *p < 0.05; **p < 0.01.

Anti‐MCPyV oncoproteins IgGs were longitudinally evaluated in sera from 3 MCC patients at baseline (T_0_) and 6th month (T_1_) of follow‐up (Figure 5). T_0_ samples of patients 1, 2 and 3 were collected 56 days from tumour radicalization, at recurrence after radiotherapy and 25 days from surgery, respectively. All patients resulted as MCPyV‐seropositive. Patient 1 exhibited a significant increase in ODs from T_0_ (0.7) to T_1_ (1.7) with LT2 (*p* < 0.05), while a slight, but not significant, increase has been observed with the remaining peptides (*p* > 0.05). This patient experienced a tumour recurrence 1 year after T_0_. Patient 3 exhibited a significant increase in ODs from T_0_ (0.5) to T_1_ (1.1) with LT1 (*p* < 0.05). A slight, but not significant, increase has been observed with the remaining peptides. At T_1_, the patient exhibited a probable partial, remission or stable disease. The IgG ODs of patient 2 were similar between T_0_ and T_1_ with all peptides (*p* > 0.05). This patient had a partial remission of the tumour after 4 cycles of therapy at T_1_.

## DISCUSSION

Herein, a reliable immunoassay with synthetic peptides was (i) computationally designed for the detection of IgGs to MCPyV LT/sT oncoproteins, (ii) experimentally developed on control sera and (iii) validated on sera from healthy individuals and MCC patients.

Computational analyses were performed to assess the reliability of linear peptides as mimotopes/antigens of MCPyV LT/sT for the detection of anti‐oncoprotein IgGs. Peptides' a.a. sequences were 100% concordant with that of MCPyV LT/sT native strains, while sequence homologies with additional PyVs were below 30%. Peptides' a.a. chains are localized within MCPyV LT/sT and environmentally exposed, suggesting that they might represent natural antigenic determinants for immune reactions. The integrative approach of sequence‐based epitope computational prediction confirmed this assumption. With the exclusion of a portion of one sT peptide, a.a. sequences of all designed peptides were predicted as specific B‐cell epitopes (Jespersen et al., 2017). B cells are well‐known to play a pivotal role in pathogen‐specific host immunity by producing antibodies against epitopes of target proteins (Liu et al., 2024). Designed peptides might therefore meet the requirement of mimotopes/antigens for detecting antibodies to MCPyV LT/sT.

Immunoassay performance was evaluated on MCPyV‐positive/−negative control sera which have been previously tested for anti‐oncoprotein antibodies with a highly reliable previously developed gold standard method (Cabán‐Hernández et al., 2014; Gardner & Greiner, 2006; Samimi et al., 2016; Swets, 1988). Numerous properties employed for determining the diagnostic performance of techniques under development were computed and interpretative criteria defined. Peptides demonstrated adequate/sufficient sensitivity, specificity, validity and PPVs/NPVs (Tankaew et al., 2017), with a good agreement between tested and expected data. *J*, *k*, LR^+^ and LR^−^ values were acceptable (Gardner & Greiner, 2006). ROCs and AUCs, which are key evaluating tools in clinical medicine were calculated (Cabán‐Hernández et al., 2014; van Gorkom et al., 2020) and, excluding sT3, AUCs of all peptides were within the low/moderately accurate ranges. The immunoassay is therefore likely capable of distinguishing MCPyV‐positive from ‐negative samples with adequate sensitivity and specificity (Gardner & Greiner, 2006; Cabán‐Hernández et al., 2014; van Gorkom et al., 2020). Moreover, correlation analysis indicated that peptides can be simultaneously employed. The assay also proved to be repeatable (Cabán‐Hernández et al., 2014), reproducible (Chandra et al., 2020), and thus precise (Bhadricha et al., 2019). Although the immunoassay exhibited lower sensitivity and specificity compared to the gold standard method (Samimi et al., 2016), performance properties suggest that the novel assay reaches acceptable prerequisites for detecting circulating anti‐MCPyV oncoprotein antibodies in human sera (Gekeler et al., 2002; Hunsperger et al., 2009; Mekonnen et al., 2021; Tankaew et al., 2017).

In this study, the development of the immunoassay provided its validation on sera from healthy individuals with unknown MCPyV serology. IgGs recognizing MCPyV LT and sT antigens were detected at a general low rate (2%–5%). A few studies, in toto conducted with viral recombinant proteins for immunoreactions, documented that the immune response against MCPyV oncoproteins in the healthy population is rare (~1%) (Paulson et al., 2010, 2017; Samimi et al., 2016). Our data therefore confirm that the immune system of healthy individuals is not exposed to LT/sT antigens. It is worth noting that previous studies documented high serological responses to MCPyV capsid proteins in different healthy populations, whose rates variate widely across studies, ranging from 46% to 68% in adults/elders (Carter et al., 2009; Csoboz et al., 2020; Sadeghi et al., 2012; Touzé et al., 2010, 2011; Van Der Meijden et al., 2013) and even 3%–87% in children/adolescents (Cason et al., 2018; Mazziotta, Lanzillotti, Govoni, et al., 2023; Nicol et al., 2013; Sourvinos et al., 2015; Van Der Meijden et al., 2013). Although these variating rates, previous studies concordantly imply that MCPyV evokes an asymptomatic infection in humans (National Toxicology Program, 2016; Touzé et al., 2010). The low rates determined herein are accountable for the scarce availability of LT/sT to trigger antibody responses given their low expression, in asymptomatically infected cells, and further nuclear translocation (Paulson et al., 2010; Tabachnick‐Cherny et al., 2020).

In conditions of persistent LT/sT expression and resulting stimulation of effector B cells, the higher is the antigen burden increase the higher is the immune response (Paulson et al., 2010; Tabachnick‐Cherny et al., 2020). Given the intrinsic structural function of capsid proteins, they are instead accessible for stimulating the humoral immune system during MCPyV asymptomatic infection conditions (Paulson et al., 2010). Hence, evaluating the immunological response to MCPyV oncoproteins in patients or individuals at risk of developing MCPyV‐driven MCC could offer valuable clinical application. Our data point in favour for the absence of a MCPyV replication/oncogenic activity in healthy individuals.

MCC is a rare tumour, whose incidence ranges 0.1–1.6 cases/100,000 individuals/year globally (Mohsen et al., 2024). As a preliminary approach, we next tested the potential of the immunoassay in longitudinally assessing the immune response to MCPyV LT/sT in sera from 3 MCC patients. Patients tested positive with all peptides, thus confirming that MCC patients harbour circulating IgGs to MCPyV oncoproteins (Paulson et al., 2010, 2017; Samimi et al., 2016). An increase in ODs was observed when patients experienced an incomplete tumour remission/stability of the disease compared to baseline, thus suggesting the constant production of anti‐oncoprotein antibodies which could parallel the evolution of the tumour. We confirm previous data highlighting an association between the anti‐oncoprotein antibody titre rise and MCC recurrence, while a falling titre has instead been related to positive outcomes (Paulson et al., 2010, 2017; Samimi et al., 2016). The amount of antibodies against viral oncoproteins can vary with the tumour burden compared to anti‐capsid antibodies which remain constant (Paulson et al., 2010, 2017; Samimi et al., 2016). Likely, the variation in anti‐oncoprotein antibodies might mirror a proper anti‐tumour immune response in MCC patients. Although further validation tests are required for assessing the potential clinical utility of the present immunoassay in sera of MCPyV‐driven MCC patients, evaluating circulating IgGs to viral oncoproteins in such patients during therapy might present prognostic relevance (Samimi et al., 2016).

Although the oncogenic potential of MCPyV depends on the activity of LT and sT (Li et al., 2015; Nooraei et al., 2021), current approaches for evaluating the MCPyV serology are predominantly focused on detecting anti‐capsid antibodies. Moreover, the methods available for detecting anti‐capsid/oncoprotein antibodies employ viral recombinant proteins, whose limitations can be circumvented by using synthetic peptides. In this regard, we have previously developed a method with synthetic peptides for detecting antibodies against MCPyV capsid proteins (Mazziotta, Lanzillotti, Govoni, et al., 2021, 2023; Mazziotta, Lanzillotti, Torreggiani, et al., 2021; Mazziotta, Pellielo, Tognon, et al., 2021). This method is suitable for assessing the epidemiology of MCPyV in healthy individuals. While the method outlined in this manuscript utilizes the same peptide‐based technology, it provides a notable advantage by identifying individuals/patients at risk for MCPyV‐driven MCC through the detection of anti‐oncoprotein IgG antibodies. Moreover, it might hold the potential for prognosticating MCPyV‐driven MCC patients.

This study presents some limitations. The longitudinal evaluation of MCPyV serology in sera from only three MCC patients is the major limit of this study. The lack of adequate sample size is mainly due to cancer rarity. Therefore, the longitudinal evaluation we carried out in this study is intended to be preliminary, while the extension of larger patients' sample size will be considered, hopefully promoting a multicenter study. Moreover, as not all MCPyV‐driven MCC patients produce antibodies against oncoproteins, while ~20% MCC is MCPyV‐negative, the larger patients' sample size should include all MCC types, i.e. MCPyV‐driven MCC expressing or not viral oncoproteins and MCPyV‐negative. An additional limitation provides the fact that anti‐capsid antibodies were not investigated in MCC sera. However, the presence of such antibodies has broadly been demonstrated in humans, and considering previous findings their evaluation might present a relatively poor clinical utility (Paulson et al., 2010, 2017; Samimi et al., 2016).

In conclusion, a novel immunoassay was developed for detecting serum IgGs against MCPyV LT and sT oncoproteins. Immunoassay performance properties were thoroughly estimated in MCPyV‐positive/−negative control sera, resulting as adequate. Assay reliability was demonstrated by studying the presence of circulating IgGs to MCPyV oncoproteins in healthy individuals and MCC patients. While the presence of circulating IgGs against MCPyV oncoproteins in healthy individuals is unlikely, preliminary evaluation of MCPyV serology in MCC patients suggests that serum anti‐oncoprotein IgGs can mirror tumour burden in such patients. However, further validations in additional sera from MCC patients are required to confirm these findings. This study represents a significant advance in evaluating the immune response to MCPyV oncoproteins in healthy individuals and MCC patients.

## EXPERIMENTAL PROCEDURES

### Sera

Controls (*n* = 256) consisted of sera from MCPyV‐positive MCC patients (*n* = 128, mean age ± standard deviation [SD], 75 ± 9 years) and MCPyV‐negative healthy subjects (*n* = 137, 60 ± 8 years), previously tested for anti‐oncoprotein antibodies with a gold standard method (Samimi et al., 2016). Additional sera with unknown MCPyV serology from healthy individuals (*n* = 100, 33 ± 7 years) and from MCC patients (*n* = 3, 79 ± 3 years), were included. MCC blood samples were collected at baseline (T_0_), i.e., after tumour excision, and at the 6th month (T_1_) of follow‐up. Written informed consent was obtained. This study complies with the Declaration of Helsinki guidelines and was performed according to ethics committee approval of the County Ethical Committee, Ferrara, Italy (ID:151078).

### Peptides and computational analyses

Computational analyses were performed to assess the reliability of LT1, LT2, LT4 and sT1 and sT3 synthetic peptides for detecting circulating IgG antibodies to MCPyV LT and sT oncoproteins (Table 3). Sequence analyses were performed with NCBI database and Clustal Omega (Hinxton, Cambridgeshire, UK). Peptides' a.a. sequences were mapped onto MCPyV LT and sT native strains to verify similarities in terms of sequence, structure and three‐dimensional (3D) conformation. The molecular form prediction/visualization of native MCPyV LT/sT strains were performed via DNASTAR (Lasergene, Madison, USA). Linear B‐cell epitope prediction was performed on LT (UniProt ID: B6DVW7_9POLY) and sT (UniProt ID: B0G0V7_9POLY) a.a. sequences by using the BepiPred Linear Epitope Prediction 2.0 tool (http://tools.iedb.org/bcell/) (Jespersen et al., 2017). The tool utilizes deep neural networks using data derived from Immune Epitope Database (IEDB). A score of 0.5 was used as threshold value for epitopes selection.

**TABLE 3:** Chemical characteristics of LT1, LT2, LT4, sT1 and sT3 synthetic linear peptides for detecting IgG antibodies elicited against Merkel cell polyomavirus (MCPyV) LT and sT oncoproteins.

Name & sequence	Nonpolar a.a. N/Tot (%)	Polar a.a. (uncharged) N/Tot (%)	Electrically charged a.a. N/Tot (%)	Electrically uncharged a.a. N/Tot (%)	Aromatic a.a. N/Tot (%)
LT1, NH_2_ – KFKEWWRSGGFSFGKAYEYGP – COOH	5/21 (24)	3/21 (14.3)	4/21 (19)	2/21 (10)	7/21 (33)
LT2, NH_2_ – ASRGAPSGSSPPHSQSSSSGY – COOH	5/21 (24)	13/21 (62)	2/10 (10)	0/21 (0)	1/21 (10)
LT4, NH_2_ – GTWEDLFCDESLSSPEPPSS – COOH	3/20 (15%)	10/20 (50)	0/20 (0)	5/20 (25)	2/20 (10)
sT1, NH_2_ – QSGYNARFCRGPGCMLKQL – COOH	7/10 (37%)	7/19 (37)	3/19 (16)	0/19 (0)	2/19 (11)
sT3, NH_2_ – SKCACISCKLSRQHCSLKT – COOH	4/19 (21%)	10/19 (53)	5/19 (26)	0/19 (0)	0/19 (0)

Abbreviations: a.a., amino acids; *N*, number.

### Immunoassay procedure and performance evaluation

The indirect immunoassay procedure was performed as detailed in Appendix S1 (Mazziotta, Lanzillotti, Govoni, et al., 2023; Mazziotta, Pellielo, Tognon, et al., 2021). Peptides' cutoffs were determined in each experiment run, as the optical density (OD) readings mean of 3 negative control sera plus 3 standard deviations of mean (mean + 3 SDs), as previously described. The immunoassay performance was evaluated on control MCPyV‐positive/−negative sera, by considering numerous conditions (Appendix S1). Moreover, Cohen's Kappa value (*k*) was used to estimate the tested/expected results agreement. Distinct peptide's performances were analysed by receiver operating characteristic (ROCs) curves, and estimation of the area under the curve (AUC, value range 0.5–1) (Swets, 1988; Cabán‐Hernández et al., 2014; Gardner & Greiner, 2006; van Gorkom et al., 2020).

### Statistical analysis

Two‐sided chi‐square/Fisher's exact tests were applied to statistically analyse seroprevalences (Vincenzi et al., 2021). ODs were analysed with the D'Agostino–Pearson normality test, while parametric/non‐parametric tests were applied according to normal/non‐normal variables (Corazza et al., 2020; Malagutti et al., 2020). Data were analysed with one‐way Anova analysis and/or Kruskal‐Wallis multiple comparison test (OD mean/median, 95% CI). Analyses were carried out using MedCalc v.16.2.1 (MedCalc, Ostend, Belgium) and GraphPad Prism v.8.0 (GraphPad, La Jolla, USA) (Mazziotta, Pellielo, Tognon, et al., 2021). Spearman correlation matrix analysis was used to evaluate the OD concordance among peptides. Linear regression of correlation coefficient (*R* ^2^) (with 95% CI) was calculated for evaluating the immunoassay accuracy. *p* < 0.05 was considered statistically significant.

## AUTHOR CONTRIBUTIONS

**Chiara Mazziotta:** Methodology; software; formal analysis; investigation; validation; writing – original draft; supervision. **Giada Badiale:** Investigation; validation. **Christian Felice Cervellera:** Methodology; visualization. **Giulia Tonnini:** Investigation; validation. **Milena Oimo:** Investigation; validation. **Antoine Touzé:** Resources; data curation; writing – review and editing. **Françoise Arnold:** Resources. **Stefania Zanussi:** Resources; data curation; writing – review and editing. **Ornella Schioppa:** Resources; data curation; writing – review and editing. **Giuseppe Fanetti:** Resources; supervision. **Mauro Tognon:** Writing – review and editing; conceptualization; supervision; project administration. **Fernanda Martini:** Writing – review and editing; supervision; funding acquisition. **John Charles Rotondo:** Conceptualization; writing – review and editing; supervision; project administration; funding acquisition.

## FUNDING INFORMATION

This work was supported, in part, by grants Associazione Italiana per la Ricerca sul Cancro (AIRC) grant 21956 (to John Charles Rotondo), and the University of Ferrara, Fondo di Ateneo per la ricerca (FAR) grants 2021 (to Fernanda Martini). J.C.R. was supported by the Umberto Veronesi Foundation with a ‘Post‐doctoral Fellowship 2023’. C.M. was supported by an AIRC fellowship for Italy (ID: 26829) and by “Bando Giovani anno 2022 per progetti di ricerca finanziati con il contributo 5 × 1000 anno 2020.”

## CONFLICT OF INTEREST STATEMENT

The following authors declare that data from this study have been employed, in part, for the Italian patent application number 102023000024003: Chiara Mazziotta, Giada Badiale, Christian Felice Cervellera, Mauro Tognon, Fernanda Martini and John Charles Rotondo. The remaining authors declare no competing interests.

## ETHICS APPROVAL STATEMENT

This study protocol was reviewed and approved by the County Ethical Committee, Ferrara, Italy, approval number [ID:151078].

## Supporting information


Appendix S1

## Data Availability

All relevant data are within the article and its Appendix S1.
