# Supplementary material for: Serum antibodies against mimotopes of Merkel cell polyomavirus oncoproteins detected by a novel immunoassay in healthy individuals and Merkel cell carcinoma patients
Source: Microb Biotechnol. 2024 Oct 25;17(10):e14536. doi: 10.1111/1751-7915.14536 (PMC11511775; doi:10.1111/1751-7915.14536)
Supplement: Supplementary file 1 — Appendix S1 [file MBT2-17-e14536-s001.docx]

*Immunoassay procedure and performance evaluation*

Immunological plates were (i) coated with 5 μg/well of the selected synthetic peptide (Thermo, Milan, Italy), (ii) diluted in 100 μL of Coating Buffer, (iii) left at 4◦C for 16 h, washed three times with Washing Buffer (WB), (iv) blocked by using 200 μL/well of blocking solution at 37°C for 90 min, (v) rinsed three times with WB and covered with 100 μL of sera diluted 1:20 in low cross-buffer (LCB) (all reagents: Candor, Wangen, Germany), (vi) incubated at 37°C for 90 min, and rinsed three times with WB, before adding a diluted 1:10,000 in LCB goat anti-human/anti-rabbit IgG heavy/light chain specific peroxidase-conjugate secondary antibody (Merck, Darmstadt, Germany). Upon incubation at room temperature for 90 min, wells were rinsed three times with WB, and then 100 μL of 2,2′-azino-bis-3-ethylbenzthiazoline-6-sulfonic acid (ABTS, Sigma, Milan Italy) was added. The intensity of color reaction was measured *via* spectrophotometer (Multiskan EX, Vantaa, Finland) at a wavelength of 405 nm. Optical density (OD) readings correspond to the extent of the immune complexes formed through the binding between the antibody and a specific peptide. Peptides’ cutoffs were determined in each experiment run, as the OD readings mean of 3 negative control sera plus 3 standard deviations of mean (mean +3 SDs).

The immunoassay performance was evaluated on control MCPyV-positive/-negative sera. The following conditions were considered: true positive (TP, number of samples positive with previous analyses), false positive (FP, number of samples positive with our assay and negative with previous analyses), true negative (TN, number of samples negative with previous analyses) and false negative (FN, number of samples negative with our assay and positive with previous analyses). Then, intrinsic characteristics of tests were computed: sensitivity (Se, TP/[TP+FN]), specificity (Sp, TN/[TN+FP]), positive and negative predictive values (PPV, TP/[TP+FP], and NPV, TN/[TN+FN]), validity ([Se+Sp]/2), accuracy (Se*Prevalence+Sp*[1−Prevalence]), overall efficienty (Ef) ([TP+TN]/[TP+TN+FP+FN] [54]. Youden’s Index (J, Se+Sp-1) and positive/negative likelihood ratios (LR+, as Se/[1-Sp] and LR-, as [1-Se]/Sp) were also evaluated. Cohen’s Kappa value (k) was used to estimate the tested/expected results agreement. K values were interpreted as poor (κ≤0), slight (0<κ≤0.20), fair (0.21<κ≤0.40), moderate (0.41<κ≤0.60), substantial (0.61<κ≤0.80), and near-perfect agreement (0.81<κ≤1.0).
